# Patterns of Subnet Usage Reveal Distinct Scales of Regulation in the Transcriptional Regulatory Network of *Escherichia coli*

**DOI:** 10.1371/journal.pcbi.1000836

**Published:** 2010-07-01

**Authors:** Carsten Marr, Fabian J. Theis, Larry S. Liebovitch, Marc-Thorsten Hütt

**Affiliations:** 1 Institute for Bioinformatics and Systems Biology, Helmholtz Zentrum München - German Research Center for Environmental Health, Neuherberg, Germany; 2 Institute for Mathematical Sciences, Technische Universität München, Garching, Germany; 3 Center for Complex Systems and Brain Sciences, Florida Atlantic University, Boca Raton, Florida, United States of America; 4 Computational Systems Biology, School of Engineering and Science, Jacobs University Bremen, Bremen, Germany; MRC Laboratory of Molecular Biology, University of Cambridge, United Kingdom

## Abstract

The set of regulatory interactions between genes, mediated by transcription factors, forms a species' transcriptional regulatory network (TRN). By comparing this network with measured gene expression data, one can identify functional properties of the TRN and gain general insight into transcriptional control. We define the subnet of a node as the subgraph consisting of all nodes topologically downstream of the node, including itself. Using a large set of microarray expression data of the bacterium *Escherichia coli*, we find that the gene expression in different subnets exhibits a structured pattern in response to environmental changes and genotypic mutation. Subnets with fewer changes in their expression pattern have a higher fraction of feed-forward loop motifs and a lower fraction of small RNA targets within them. Our study implies that the TRN consists of several scales of regulatory organization: (1) subnets with more varying gene expression controlled by both transcription factors and post-transcriptional RNA regulation and (2) subnets with less varying gene expression having more feed-forward loops and less post-transcriptional RNA regulation.

## Author Summary

Bacterial cells can adapt to various genomic mutations and intriguingly many environmental changes. They do this by adjusting their gene expression profile to meet the requirements of a new condition. In this work, we study the interplay of different mechanisms of gene regulatory control driving this adaptation in the bacterium *E. coli*. We deconstruct the network of all transcription factor mediated regulatory interactions into subnets, topologically defined subgraphs which we expect to act as information processing units. Indeed, we find that many subnets react coordinately to cellular stress, and are used by the cells to account for mutations. In these subnets, we also find many small RNA targets. In contrast, those subnets that do not act in a coordinated fashion are highly enriched with feed-forward loops, a 3-node network motif with important information processing properties. Our approach reveals correlations and anti-correlations of three scales of regulatory control: subnets, feed-forward loops, and small RNA.

## Introduction

An interesting topological feature of the transcriptional regulatory network (TRN) of the bacterium *Escherichia coli* is its almost tree-like structure with only few loops (see [1] for a detailed discussion and comparison with the TRN of the yeast *Saccharomyces cerevisiae*). This observation has several consequences. First, hierarchical levels in the network can be meaningfully defined and analyzed. Second, it leads to the question, on which level of organization information processing takes place in the TRN given a dominant directed flow dictated by the network's architecture. On a local scale, substructures in the TRN that appear significantly more often than in corresponding randomized networks—so-called network motifs [2], [3]—have been found to match specific information processing steps. Particularly feed-forward loops have been theoretically proposed [4] and experimentally supported [5], [6] to function as noise-suppression units and delay devices.

**Figure 1:**
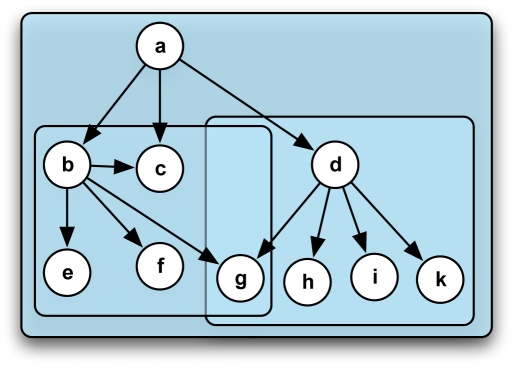
Illustration of the subnet approach. A subnet is defined as the subgraph induced by all nodes downstream of a root node, including the root node. The network in this figure contains three subnets: the subnet of root **a** comprises all nodes in the network, organized in three hierarchical layers. The subnet of root **b** contains **b** and all downstream nodes **c**, **e**, **f**, **g**. The subnet of root **d** contains **d**, **g**, **h**, **i**, **k**. Notably, the subnet of root **a** contains a feed-forward loop formed by nodes **a**, **b**, and **c**, while the subnet of root **d** constitutes a single input motif.

Here we dissect the TRN into topological modules. We define the subnet of a node (root) as the subgraph consisting of all nodes topologically downstream of the root, including the root node itself (see Figure 1 for an illustration of the concept). Subnets can extend over multiple hierarchical layers if they contain a hierarchy of transcriptions factors (TFs). Moreover, they can overlap if genes are regulated by TFs from different subnets. Some network motifs such as the feed-forward loop or the single input motif are subnets themselves and therefore fully contained in at least one subnet. This approach is possible due to the topological properties of the *E. coli* TRN: apart from the few small cycles in the network (see Results), most subnets are directed acyclic graphs.

The search for the imprint of the transcriptional regulatory network in gene expression profiles is a search for very weak signals, often masked by the broad range of additional biological processes (beyond the regulation via transcription factors) shaping the expression of a gene. In two previous studies [7], [8], the consistency between expression profiles and pairwise interactions in the TRN has been shown to be surprisingly low. The consistency on a larger scale has been studied for a specific type of subnets, named ‘origons’ [9]. There, the authors find that genes in some origons are selectively affected by specific environmental signals. In this contribution, we study patterns of subnet usage for two markedly different genome-wide gene expression data sets. As is [9], we use microarray expression profiles from the ASAP database, where wild-type expression under standard growth conditions is compared to a variety of profiles with external stimuli and genetic alterations. As a second data set, we use the time-course data of [10]. Here, *E. coli* strains are exposed to different media and stresses, and profiled at up to 16 time points. We analyze subnets with respect to their responsiveness to altered conditions in both data sets and classify them according to the observed subnet usage patterns.

*E. coli* employs different scales of regulatory control to establish homeostasis (see, e.g., [11]) or to adapt to external stimuli. Recently, we introduced the concept of digital and analog control to differentiate between the regulatory response coordinated by dedicated TFs and DNA architectural proteins, respectively [12]. We found that as soon as one form is limited (by TF mutations or changes in the DNA superhelicity), the other form of control compensates, exhibiting a balance of regulatory control. An analysis employing methods from point process statistics has been able to further support the interplay of digital and analog control by analyzing gene distributions [13]. In the following, we want to delineate the interplay between the subnet usage as a TF mediated, topologically based form of control, and two other scales of regulatory control: translational inhibition and mRNA degradation induced by small non-coding RNAs (sRNAs) and the dynamic coordination of nodes connected in a feed-forward loop.

## Results

### Networks

We consider the most complete prokaryotic TRN available, the TRN of the bacterium *E. coli*. Nodes in our network correspond to genes (and the respective TF) while a directed edge represents a regulatory interaction mediated by a TF. Based on the version 6.3 of the Regulon database [14], the TRN comprises 1515 nodes and 3171 links, with 162 regulators (i.e. nodes which regulate at least one other gene) and 1432 target nodes (i.e. nodes which are regulated by at least one other gene).

**Figure 2:**
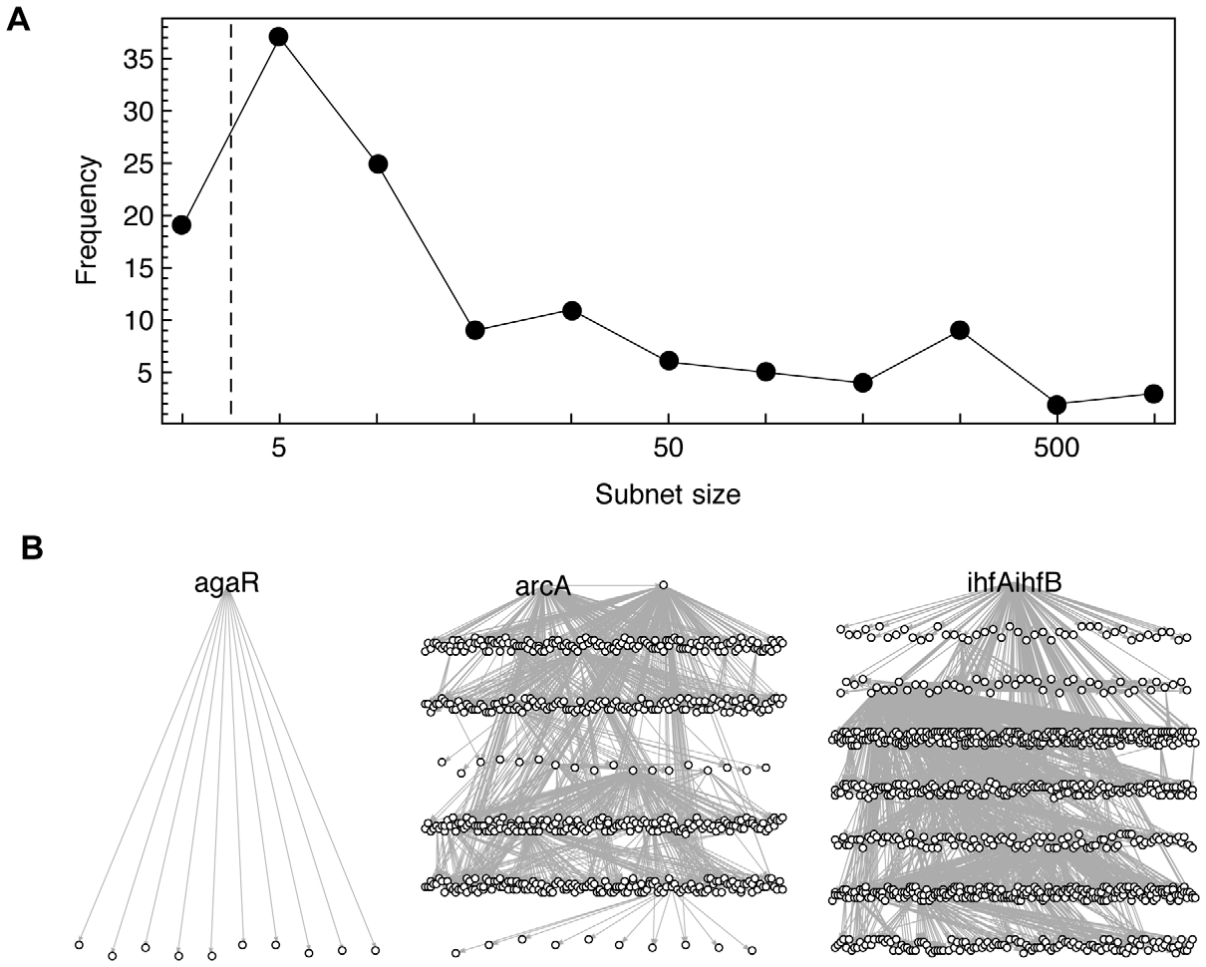
Subnets in the transcriptional regulatory network (TRN) of *E. coli*. The TRN can be decomposed into subnets, defined by the root node, comprising all nodes topologically downstream. (A) Histogram of subnet sizes, binned on a logarithmic scale. We only consider subnets of five nodes or more to allow for significantly enriched subnets. (B) The *ihfAihfB* subnet is the largest subnet in the TRN, comprising 1021 nodes organized in seven hierarchical levels. The transcription factors *arcA* and *fnr* regulate each other and therefore share 650 downstream nodes. The *agaR* subnet has only 11 nodes, organized as a single input motif.

We dissect the TRN into subnets, defined as subgraphs consisting of a root node with at least one regulatory interaction, and all downstream nodes (see Figure 1A for an illustrative example network consisting of three subnets). The 162 subnets of the TRN are overlapping and of very different sizes and hierarchical complexities (see the frequency distribution of subnet sizes in Figure 2A and the histogram of relative subnet overlap in Figure 2 in Text S1). Let us consider three examples: the *ihfAihfB* subnet (see Figure 2B and Figure 1 in Text S1 for a highly resolved version) is the largest subnet in the *E. coli* TRN with 1021 downstream nodes, among them many regulators, organized in seven hierarchical levels. For the genes *ihfA* and *ihfB*, we consider only one subnet, since their regulatory action is mediated by the IHF hetero-dimer, formed by the gene products of both genes. In contrast to older versions of RegulonDB, release 6.3 contains eight mutual interactions between gene pairs, and one 3-node cycle (see Materials and Methods), leading to subnets with many shared downstream nodes (as shown in Figure 2B for *fnr-arcA*). An exemplary small subnet is shown for the TF *agaR* in Figure 2B. It contains no regulators and can thus be depicted as a tree with only two hierarchical levels: the root node *agaR* at the top and all ten target nodes in the bottom layer.

### Subnet usage

**Figure 3:**
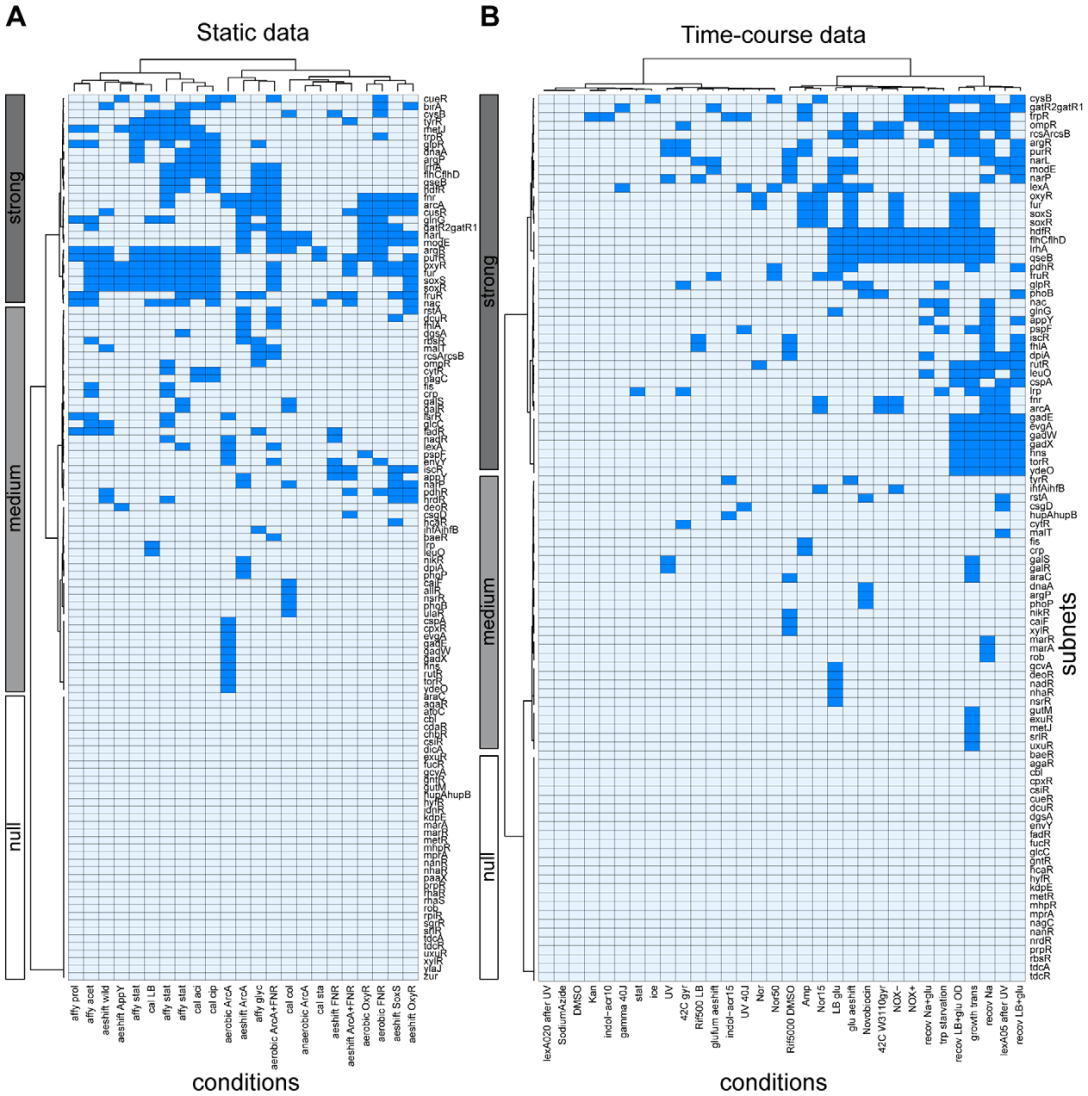
Subnet usage matrices. The subnet usage matrix consists of subnets (rows) and conditions (columns) for the static ASAP data (A) and the time-course data of Sangurdekar et al. [10] (B). A deep blue entry represents a subnet significantly enriched with differentially expressed genes under the respective conditional change (A), or a subnet with collectively responding genes during the given time-course (B), respectively.

We want to analyze the importance of subnets as information-processing units in the TRN. To this end, we map large-scale expression profiles from microarray experiments onto the TRN. First, we consider a data set where either wild-type *E. coli* strains are compared to strains with genetic alterations and with cells under environmental stress, or wild-type and mutant strains are compared under aerobic and anaerobic growth conditions. We will refer to this data set as the static data (see Materials and Methods for a detailed description of the data used). For each condition, we identify differentially expressed genes (with a statistical analysis of microarrays as introduced in [15], 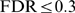, see Materials and Methods) and determine subnets significantly enriched (Fisher's exact test at 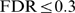, see Materials and Methods) with those genes. In Figure 3A, we plot a hierarchically clustered (see Materials and Methods for clustering details) subnet usage matrix, where a deep blue entry represents a subnet significantly enriched with differentially expressed genes.

For example, the comparison of wild-type and *fnr* mutant strains under aerobic growth conditions (denoted as ‘aerobic FNR’ in the usage matrix labels) yields 17 subnets with enriched differentially expressed members: *arcA*, *argR*, *birA*, *cueR*, *cusR*, *cysB*, *envY*, *fnr*, *fur*, *gatR2gatR1*, *glnG*, *modE*, *narL*, *oxyR*, *pdhR*, *purR*, *trpR*. We assume that these subnets are directly associated with the *fnr* deletion, either due to the TF action of FNR (the roots *arcA*, *narL*, and *pdhR* are direct targets of FNR) or via signal transduction cascades induced by the presence or absence of FNR. Interestingly, not all subnets embedded in the *fnr* subnet show significant differences in the expression of their genes. These phenomena may occur due to missing data in RegulonDB or due to interactions that rely on specific conditions and are not active under aerobic growth (like co-activators or TF conformations). A functional hypothesis is that the downstream genes of the respective root are collectively shielded from the rest of the *fnr* subnet, or that the regulatory control of the respective node exceeds the pure promoter binding mechanism (as the analog control [12] of the known architectural protein H-NS, see also e.g. [16]).

The overall pattern of subnet usage for the different conditions is rather homogeneous for all compared profiles: between 6.8% and 20% of the subnets are used in each condition. However, we find a hierarchy of usage at the subnet level and coordinately used subnets. A clustering of subnets with respect to their subnet usage will be discussed in the next section.

We want to compare our results with another, fundamentally different data set consisting of time series, and an independent analysis approach based on the collectivity of a subnet's response. The data used in [10] contains time courses of *E. coli* transcriptome responses to diverse stimuli (like UV and gamma radiation, norfloxacin, and different concentrations of indol-acrylate), measured with whole-genome DNA microarrays. For each time series we quantify the collectivity of the response of the subnet's genes and compare it to randomly sampled subnets by calculating the Shannon entropy of the eigenvalues of a singular value decomposition (see Materials and Methods for details). Subnets responding collectively are marked in Figure 3B as deep blue entries.

During the different time courses, the subnet usage varies between no subnet usage at all (0%) and a maximum of 26%. The first 14 experiments in the matrix (including all radiation exposure experiments and indol-acrylate treatments in different concentrations) exhibit a subnet usage below 0.5%. Apparently, for these experiments, *E. coli* masters the adaptation to the imposed stress with other forms of regulatory control. In experiments where subnets are more frequently used, we find again blocks of collectively used subnets that differ between sets of experiments.

### Clustering

Using hierarchical clustering, we identify clusters of subnets with distinctly different patterns of subnet usage in both data sets. In the subnet usage matrix derived from the static data (Figure 3A), a substantial part of the subnets are never significantly enriched with differentially expressed genes, further on called the ‘null’ cluster. On the contrary, subnets in the ‘strong’ cluster are on average used in 25% of the experiments. The ‘medium’ cluster in between has an average usage of 6.0%. In the time-course data matrix (Figure 3B), we also identify three clusters with markedly different subnet usage, further similarly denoted as ‘strong’ (20% average subnet usage), ‘medium’ (3.8%), and ‘null’ (0.0%).

**Figure 4:**
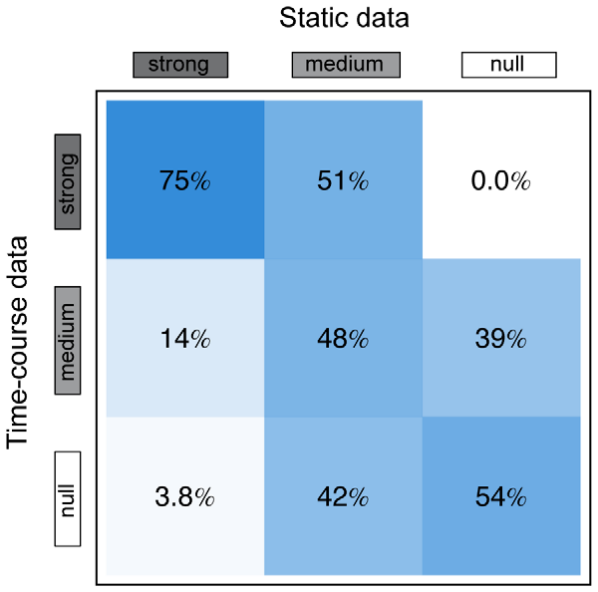
Subnet cluster overlap. Relative overlap between the different subnet clusters of the static data and the time-course data. The relative overlap is calculated as the number of subnets present in both clusters, divided by the smaller total number of subnets in the two clusters under consideration.

The overlap—with respect to the subnet roots—between clusters from the two data sets is shown in Figure 4. We find that the clusters in the different experiments often share subnets (75% for the ‘strong’, 48% for the ‘medium’, and 54% for the two ‘null’ cluster). Only some subnets in the ‘strong’ cluster of the static data appear in the ‘null’ cluster of the time-course data (3.8% overlap), the static ‘null’ cluster and the time-course ‘strong’ cluster are disjunct (0% overlap). The fact that the cluster composition differs between the two data sets may rely on the different external stresses applied. Maybe even more importantly, in the time-course data, an *E. coli* colony adapts spontaneously to a environmental change applied. In contrast, strains that have already adapted to a different environment or a genetic mutation are compared in the static data. Still, the large overlap between the clusters, derived from experiments with independently sampled environmental conditions, is remarkable.

To assess the cluster composition from a functional perspective and detect biological plausible components, we conducted a gene ontology (GO) enrichment analysis (see Materials and Methods for details). On the level of subnet roots, we find no enriched GO terms at all. If we include the nodes within the subnets in each cluster, we find several enriched categories. In the ‘strong’ clusters of the static and time-course data, ‘iron ion binding’ and the ‘generation of precursor metabolites and energy’ appears. The less overlapping ‘medium’ clusters share no enriched annotations. The ‘null’ clusters, finally, share enriched metabolic processes (carbohydrate, fucose, D-gluconate) and transporter activity (carbohydrate, sugar).

**Figure 5:**
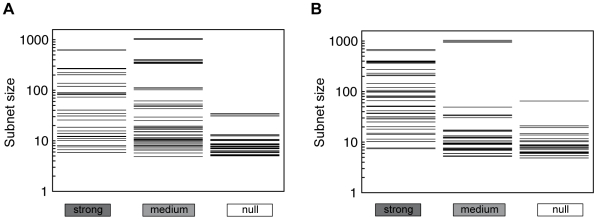
Subnet size composition of the clusters. For the static data (A) and the time-course data (B), the ‘null’ cluster is composed of subnets with less than 100 nodes, while all other clusters contain both small and large subnets.

We study the sizes of the subnets contained in the different clusters and find that the subnet composition is highly heterogeneous in both the static (Figure 5A) and the time-course data (Figure 5B): while the ‘strong’ and ‘medium’ clusters contain subnets of all size, including large subnets with hundreds of nodes spanning most of the TRN, the ‘null’ clusters contain preferentially small subnets with only tens of nodes (see Figure 5). Similarly, the out-degrees of the subnet roots substantially differ. While the master regulators *fnr* in the ‘strong’ and *crp* in the ‘medium’ cluster control 275 and 418 nodes, respectively, the maximum out-degree of ‘null’ cluster subnets is 28 for *marA* in the static case, and 57 for *cpxR* in the time-course data.

### Motifs

**Figure 6:**
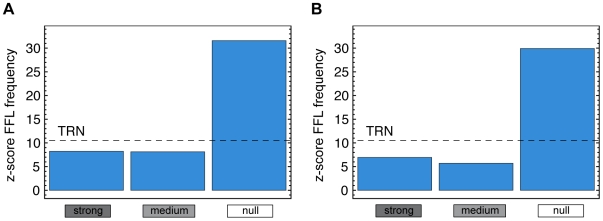
Feed-forward loop enrichment. Analysis of the feed-forward composition of the subnet clusters identified in the static data (A) and the time-course data (B) respectively. In both data sets, we find that the z-score of the feed-forward loop composition is highest in the ‘null’ cluster induced by non-responding subnets.

Can we infer topological differences between the conditionally used subnets and the unused subnets in the ‘null’ cluster beyond subnet size and a root's out-degree? We analyzed the 3-node motif composition of the subgraphs induced by the subnets of each cluster (see Figure 3 in Text S1) by computing the z-score (see Materials and Methods for a detailed description of the z-score calculation) with respect to randomized graphs [2]. All subnets show a normalized triad significance profile [17] characteristic for bacterial regulatory networks (see Figure 4 in Text S1). However, consistently in both data sets we find in the ‘null’ cluster an enrichment of feed-forward loops, a well-studied motif with interesting dynamical properties (see Figure 6). Depending on the actual design as a coherent or incoherent feed-forward loop, this motif can serve as a sign-sensitive delay or an accelerator in transcriptional networks [4]. Here, the feed-forward loop z-scores of 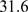 and 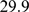 for the static data and the time-course data ‘null’ cluster, respectively, distinctly exceed the z-score of the feed-forward loop in the full TRN (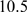). The z-scores of all other clusters lie below this threshold (see Figure 6).

To check whether enriched feed-forward loops are an artifact of the cluster-induced subgraph sizes, we apply two null models to the static data: First, we induce a subgraph of the TRN by randomly sampling the same number of nodes as contained in the ‘null’ cluster (that is, 221). Second, we randomly choose the same number of subnets as contained in the ‘null’ cluster (that is, 30) and therein induce subgraphs with a size distribution similar to the one in the ‘null’ cluster. We generate 100 samples and find that the feed-forward loop z-score of the ‘null’ cluster exceeds both null model averages (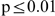 and 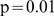, respectively, see Figure 5 in Text S1). This indicates that the feed-forward loop enrichment is a specific property of the identified ‘null’ cluster and no size effect.

We test the robustness of our finding with regard to the data used in two different ways: First, we apply a meta analysis on the 466 *E. coli* experiments available in the Many microbes microarray database [18]. We analyze this data with the entropy approach by interpreting the set of experiments as a time series. Interestingly, the homogeneously responding subnets show no distinct feed-forward loop enrichment (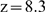) while the subnets with no coordinated response are, similarly to the ‘null’ cluster subnets, highly enriched with feed-forward loops (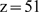). Second, to test the robustness of our findings against incomplete data, we implement the time-course data analysis on the last four version of RegulonDB (6.1–6.4). We find that irrespective of the RegulonDB version used in our analysis, a prominent feed-forward loop enrichment in the null cluster appears (see Figure 6 in Text S1). Notably, the number of vertices (V) and links (L) in the TRNs increased considerably from 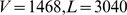 (RegulonDB 6.1) to 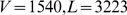 (RegulonDB 6.4).

### Small RNA target enrichment

**Figure 7:**
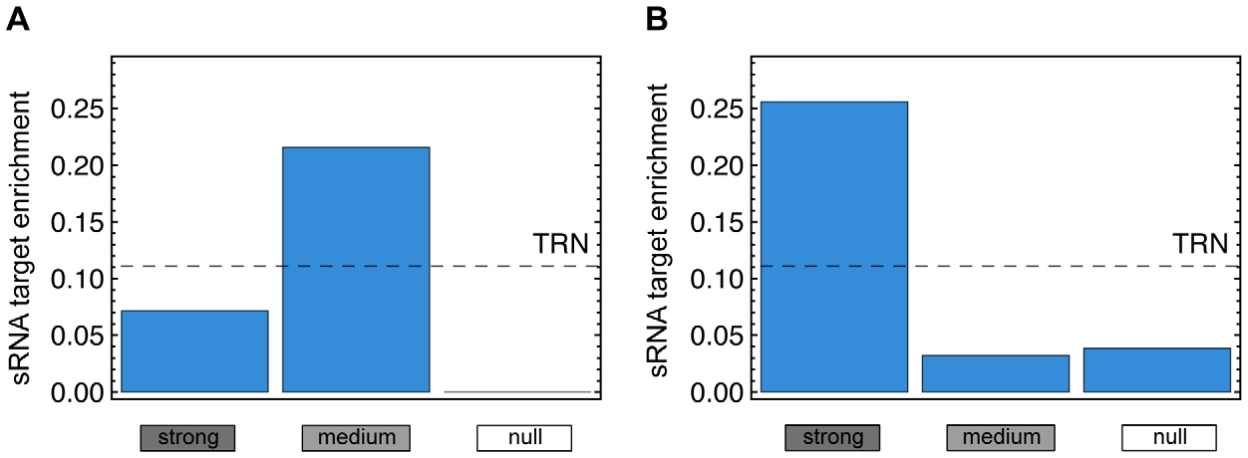
sRNA target enrichment. Relative number of subnets with enriched sRNA targets in each cluster in the static data (A) and the time-course data (B) respectively. In the full TRN, we find 13 out of 117 subnets significantly enriched with sRNA targets (Fisher's exact test with 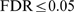), resulting in an average sRNA target enrichment of 0.11 (dashed line). In both data sets, we find that subnets in the ‘null’ cluster are depleted with sRNA targets.

A rather recently discovered mechanism of regulatory control are small noncoding RNAs (sRNAs) [19]. In *E. coli*, up to 100 sRNAs may exist [20], primarily as regulators of mRNA stability and translation. We first investigate the sRNA mediated control on network motifs. Comparing the number of 3-node motifs with at least one sRNA target with randomly sampled sets of targets of the same size, we identify seven motifs with significantly (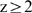) enriched occurrence of sRNA targets (see Figure 7 in Text S1). Among them, we find the feed-forward loop (motif ID 38), and a motif (ID 110, see Figure 7 in Text S1), which has been implicated previously with an enrichment of microRNA targets in a mammalian signaling network [21].

To infer the interplay between subnet mediated control and sRNA regulation, we map the target transcripts from RegulonDB 6.3 onto the TRN and infer 13 subnets with a significantly enriched (Fisher's exact test, 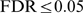, see Materials and Methods) number of sRNA target genes: *arcA*, *cspA*, *envY*, *evgA*, *fnr*, *gadE*, *gadW*, *gadX*, *hns*, *ihfAihfB*, *rutR*, *torR*, *ydeO*. In relative numbers, we find that 11% of all TRN subnets are enriched with sRNA targets. With regard to the clusters of different subnet usage, enriched subnets are intriguingly absent in the cluster with unused subnets: We find no enriched subnet in the ‘null’ cluster of the static data, and only one (*envY*) in the time-course data (see Figure 7). At the same time, enriched subnets are present in the medium and strong cluster, respectively.

We draw two important conclusions from that finding: First, the clusters inferred from the subnet usage analysis establish categories on the set of subnets that appear to have markedly different topological and regulatory properties. Second, regulatory control on the subnet level coincides with sRNA mediated control, while feed-forward loop dynamics seems less dependent of the impact of sRNA.

## Discussion

The rationale of our analysis has been to explore the internal logic of gene regulation by looking at different scales within the transcriptional regulatory network of *E. coli*. The post-transcriptional regulation mediated by sRNAs coincides with the subnet-wide control conferred by TFs. In contrast to this correlated regulatory control, we obtain an anti-correlated pattern for subnet usage and the occurrence of feed-forward loops: when the scale dominates (high subnet usage) few regulatory devices on the smaller scale are found (low feed-forward loop occurrence). Similarly to our previous data-driven study on the buffering of digital and analog control [12], our results indicate a systematic interplay between distinct regulatory mechanisms. However, in contrast to the concept of analog and digital control, there is no evidence for a balancing between the induction of subnets and the usage of feed-forward loops. Rather, from the static data analysis (see Figure 3A) we see that upon mutation of a root node of a strongly responding subnet, other subnets compensate for the compromised control. The reason for that may be the difference in scales: while both analog and digital control can operate on sets of up to hundred genes, there is a huge functional discrepancy between the genome-wide regulation of large subnets and the dedicated dynamical tuning of few nodes by a feed-forward loop.

Our study expands previous approaches to link topological properties of the TRN with expression profiles. Subnets as topologically defined units of the TRN are groups of genes that deal coordinately with conditional or environmental changes due to shared regulatory interactions. The ‘regulon’ concept, where genes are pooled if they share a common transcription factor, is extended by taking into account the full downstream regulation instead of only the first hierarchical layer. ‘Origons’ [9] are the subset of subnets with no regulatory input at the root node and have been defined on an operon-based version of the TRN (that is, genes with the same promoter are treated as one node). Based on the assumption that every TF is able to sense signals in the cell, the subnet notion is a natural generalization of the origon concept: It allows for the identification of used subnets within larger unused subnets (which may be origons) and, vice versa, small unused subnets within larger used origons.

In a complementary, subsequent investigation, one could study the sRNA target enrichment and feed-forward loop usage across the different experimental conditions, similarly to the study of [22]. This would require to distinguish the different types of coherent/incoherent feed-forward loops [6] and quantify their usage. Here we introduced the subnet notion, verified our approach with two types of large-scale expression data, and compared distinct scales of regulatory control in clusters with different subnet usage.

## Materials and Methods

**Figure 8:**
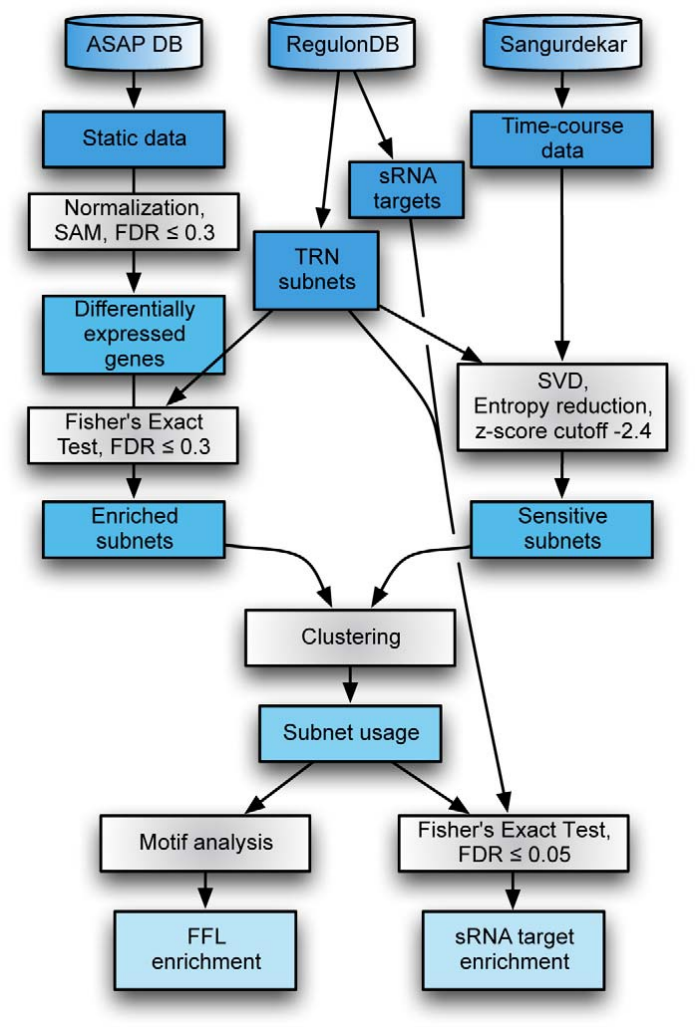
Workflow. Static data and time-course data are analyzed differently up to the identification of subnets. Clustering and motif analysis is applied similarly to the resulting subnets.

The workflow of our analysis is illustrated in Figure 8, with details as follows.

### Network data

We use the RegulonDB 6.3 [14] data on TF-gene interactions to construct the *E. coli* TRN. Dimer TFs (e.g. *flhCflhD* and the corresponding genes *flhC* and *flhD*) are merged to a single node in the network, phantom genes (i.e. a gene that at a previous time it was thought to be a gene, but more recent analyses indicate it is not) are removed. The resulting TRN comprises 1515 nodes with 3270 interactions including 99 self-loops.

### Subnet construction

Within the TRN, 162 nodes have outgoing links to other nodes. The corresponding genes confer regulatory control to other genes via TF binding, and are called roots further on. Each subnet is defined by a root node and all the nodes topologically downstream. The level of a node within the subnet is defined as the maximal distance of this node to the root node. Pairs of genes regulating each other share the same level. In Regulon 6.3, we find eight two node cycles (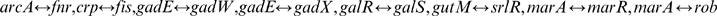) and one 3-node cycle (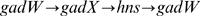). Subnets can overlap, if they share downstream nodes (see Figure 1A). To allow for significant enrichment in our expression analysis, we only consider subnets with five nodes or more (see Figure 2 for a histogram of the subnet sizes), ending up with 117 subnets out of 162 contained within the TRN. The subnets are deposited in Text S2.

### Expression data

We consider two different expression data sets to study patterns of subnet usage. As static expression profiles, we use Affymetrix chip data contained in the ASAP database (https://asap.ahabs.wisc.edu/asap/home.php) [23], namely the data sets ‘Aerobic shift’, ‘Calibrator’, and ‘Affy data’. In each data set, we compare different environmental (like ‘heat-shock’) or genotypic (like ‘fnr deletion’) conditions with the respective wildtype experiments, ending up with 39 chip comparisons. In the ‘Aerobic shift’ data set, we first calculate the estimated transcript copy numbers (ETCNs) and compare mutant strains with wildtype strains under both aerobic and anaerobic growth conditions.

For time-course expression profiles, we use data from [10]. There, *E. coli* strains are cultured and subsequently analyzed on whole-genome microarrays under diverse conditions like ‘normal growth’, ‘suboptimal growth’, ‘transient arrest’, or ‘severe arrest and killing’. We use 32 time-course data sets, the number of time points varying from experiment to experiment between 2 and 16.

### Subnet usage

Due to the different experimental setups we apply two different approaches to quantify the subnet usage.

For the static data, we first determine differentially expressed genes between two experimental conditions. For all three data sets (‘Aerobic shift’, ‘Calibrator’, ‘Affy data’), we compare a specific condition with its corresponding wild-type condition (e.g., we compare the anaerobically grown FNR deletion mutant with the anaerobically grown wild-type strain). Additionally, we regard the various mutants under aerobic and anaerobic conditions (e.g., we compare the OxyR mutant expression profiles with and without oxygen supply from the ‘Aerobic shift’ data). In each of the 33 resulting data set pairs (condition vs. wild-type and aerobic vs. anaerobic, respectively) we determine differentially expressed genes by applying the ‘Statistical Analysis of Microarrays’ (SAM) algorithm introduced in [15] with a Wilcoxon rank statistics and a False Discovery Rate 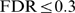. We disregard experiments with no genes below the significance level (10 out of 33). We then calculate a p-value for the enrichment (that is, a higher fraction of differentially expressed genes within the subnet as compared to the whole TRN) with Fisher's Exact Test. After multiple testing correction, we call subnets with 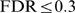 significantly enriched and mark them in dark blue in the subnet usage matrix in Figure 3A.

For the time-course data, we use a similar approach as described in [10]. To evaluate, if the genes of a given subnet respond collectively during time to the stimulus applied, we calculate the Shannon entropy 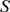 of the normalized eigenvalues 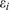 of a singular value decomposition (SVD) of the 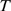 time-points vs. 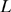 genes matrix, as described in [24]:
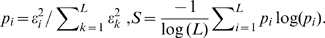



Collectively corresponding genes give rise to a dominant principal eigenvalue 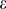 and a small 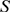. For each subnet and each time-course experiment, we randomly sample pseudo subnets (that is, we randomly choose the same number of genes as contained in the respective subnet) 1000 times and calculate a z-score (the deviation of the subnet's entropy 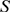 from the mean 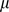 of the sampled distribution, divided by the standard deviation of the sampled distribution 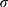, 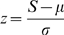). From the 117 subnets under consideration, we take only those with three or more genes included in the data set from [10], reducing the number of analyzed subnets to 100. In order to keep the overall number of insensitive subnets comparable to the static data (25%), we choose a z-score of 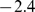 as cutoff. Collectively corresponding subnets are marked in dark blue in the subnet usage matrix in Figure 3B. We validate our results with the four latest versions of RegulonDB (6.1–6.4), where we keep the size of the ‘null’ cluster constant at 25% and adapt the z-score cutoff accordingly.

As an additional meta analysis, we take the 466 *E. coli* experiments available in the Many microbes microarray database (M3D) [18]. We analyze this data with the entropy approach by interpreting the set of experiments as a time series. Disregarding the principal value in the SVD, and thus eliminating the vast chip-wide differences between the 466 experiments included in M3D, we compare the entropy of a subnet with the entropies of randomly sampled subnets and calculate a z-score. Similarly to our previous analysis, we interpret subnets with z-scores below and above the threshold 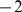 as collectively (strongly) responding and not responding (null), respectively. From these two sets of subnets, we induce subgraphs and calculate the feed-forward loop enrichment in the respective graphs. Interestingly, the strongly responding subnets show no distinct feed-forward loop enrichment (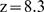) while the subnets with no coordinated response are, similarly to the ‘null’ cluster subnets, highly enriched with feed-forward loops (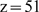). Notably, disregarding the principal value in the SVD in the analysis of the time-course data does not alter our results.

### Hierarchical clustering

From the analysis of subnet usage, we end up with two matrices, one for the static data (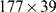) and one for the time-course data (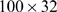). The matrices contain a 1 for a subnet significantly enriched with differentially expressed genes or significantly correlated time-courses, respectively, and a 0 otherwise. We hierarchically cluster the two matrices using the Manhattan distance function (see e.g. [25]) and the Ward agglomerative algorithm [26]. We end up with three clusters of subnets with clearly different usage patterns throughout the different experiments.

### Gene Ontology enrichment

To infer GO term overrepresentation in the different clusters of subnet usage, we use GOstat [27] with *E. coli* UNIPROT identifiers and the ‘goa uniprot’ database. As parameters of the statistical test we use a p-value cutoff of 0.01 with the Holm multiple testing correction method and a GO-Cluster Cutoff of 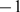.

### Motif analysis

For each cluster, we induce a single subgraph of the whole TRN by taking all nodes of the cluster's subnets. We thus ensure that every motif is counted only once in each cluster. We calculate the z-scores of the network motifs of size 3 in the TRN and the cluster induced subgraphs with MFINDER [3] (using 1000 random networks). For this analysis we disregard the character of the interaction (i.e. its activating or inhibiting impact).

### sRNA enrichment

RegulonDB 6.3 contains regulatory information for 22 small RNAs and 32 target transcripts. We map these onto the TRN and find 22 target genes, within them the roots *fhlA*, *gadX*, and *hns*. We map the targets on the TRN subnets and calculate the relative overrepresentation with Fisher's exact test. We correct for multiple testing error and find 13 subnets enriched with sRNA targets at 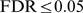: *arcA*, *cspA*, *envY*, *evgA*, *fnr*, *gadE*, *gadW*, *gadX*, *hns*, *ihfAihfB*, *rutR*, *torR*, *ydeO*. For each cluster, we calculate the relative number of subnets with sRNA target enrichment and plot the result in Figure 7.

## Supporting Information

Text S1 Text S1 contains 7 supplementary figures.
(0.95 MB PDF)

Text S2 The file contains all 117 subnets used in our study, derived from RegulonDB 6.3. The first gene in each line represents the root node of the respective subnet.
(0.05 MB TXT)
