# Supplementary material for: Patterns of Subnet Usage Reveal Distinct Scales of Regulation in the Transcriptional Regulatory Network of Escherichia coli
Source: PLoS Comput Biol. 2010 Jul 1;6(7):e1000836. doi: 10.1371/journal.pcbi.1000836 (PMC2895633; doi:10.1371/journal.pcbi.1000836)
Supplement: Text S1 — contains 7 supplementary figures. (0.95 MB PDF) [file pcbi.1000836.s001.pdf]

Patterns of subnet usage reveal distinct scales of regulation in the transcriptional regulatory network of *Escherichia coli*

Carsten Marr[1,*], Fabian J. Theis[1,2], Larry S. Liebovitch[3], Marc-Thorsten Hütt[4]

1 Institute for Bioinformatics and Systems Biology, Helmholtz Zentrum München - German Research Center for Environmental Health, Neuherberg, Germany
2 Institute for Mathematical Sciences, Technische Universität München, Garching, Germany
3 Center for Complex Systems and Brain Sciences, Florida Atlantic University, Boca Raton, Florida, USA
4 Computational Systems Biology, School of Engineering and Science, Jacobs University Bremen, Bremen, Germany
∗ E-mail: carsten.marr@helmholtz-muenchen.de

Text S1: Supplementary figures

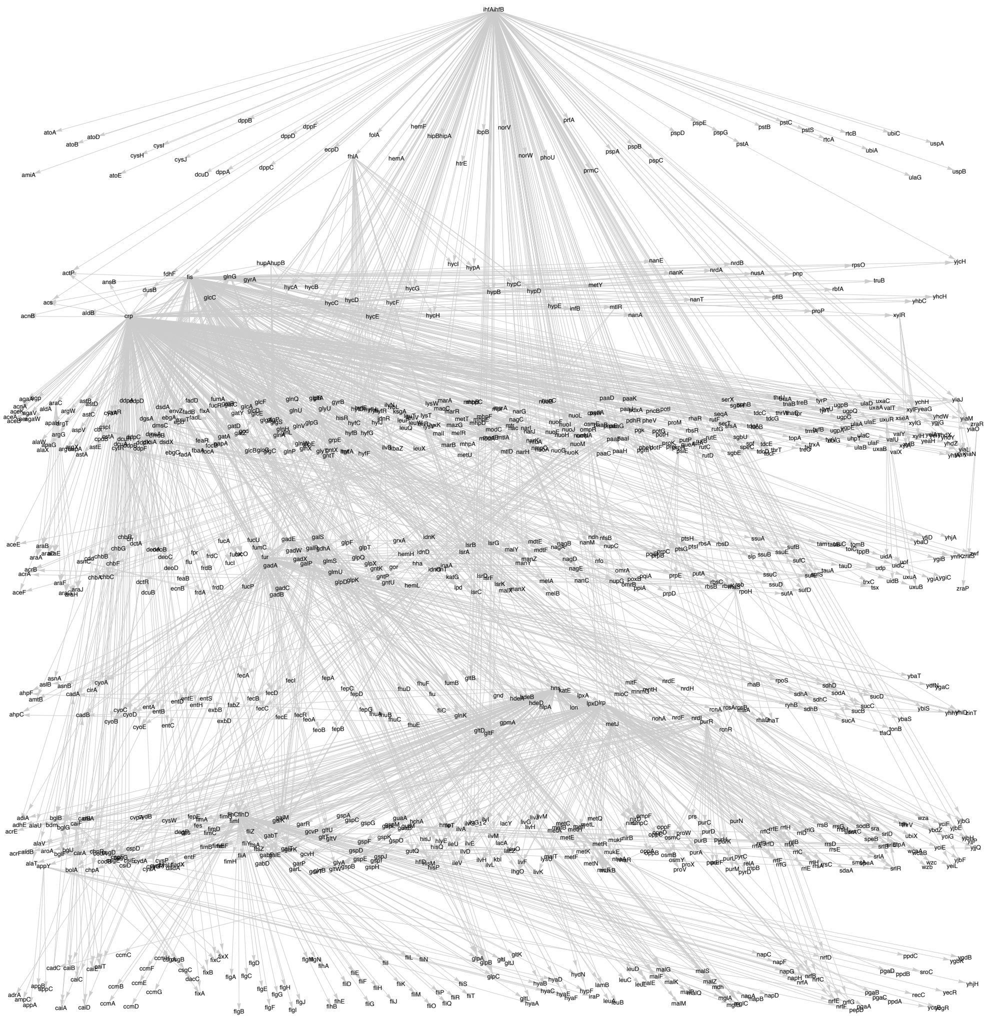

Figure 1. A highly resolved version of the ihfAihfB subnet.

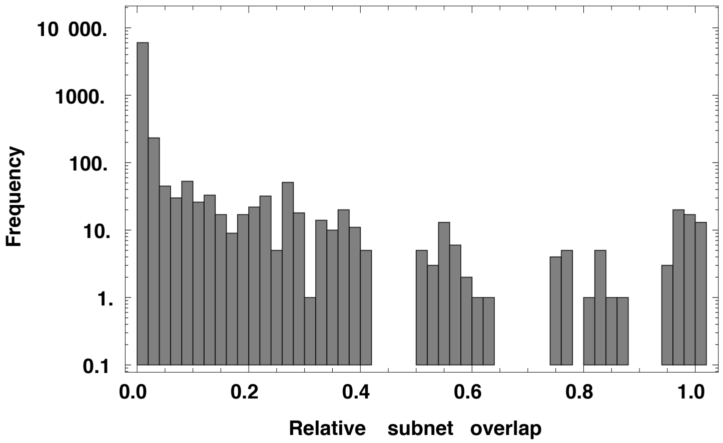

Figure 2. Histogram of the relative overlap of all pairs of subnets. We plot the frequency distribution of the relative overlap (Intersection/Maximum) calculated for all $117 \times 116/2$ pairs of subnets. Interestingly, apart from a peak at 0, the subnet overlap is a fairly equally distributed quantity.

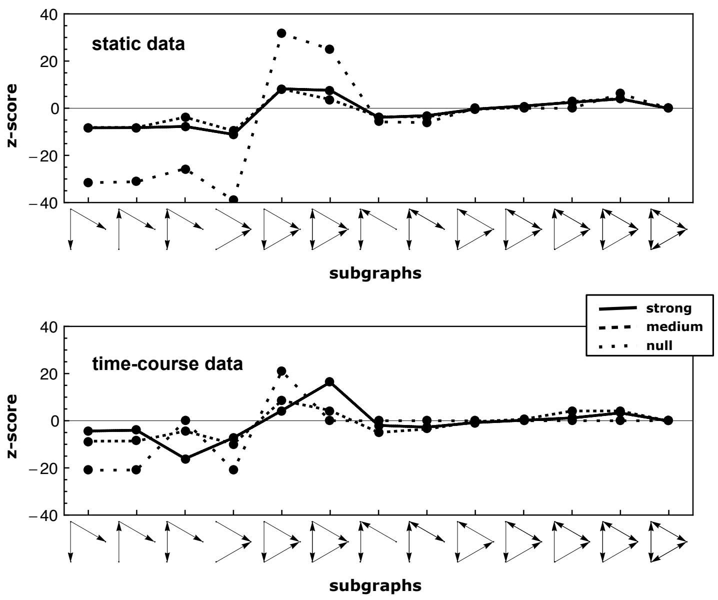

Figure 3. z-score profiles of all 13 possible directed triads. For the two expression data sets (top: static data, bottom: time-course data) we calcluate the z-score profiles for all identified clusters of different subnet usage. In both data sets, we find the feed-forward loop (5th triad) to be overrepresented in the 'null' cluster, in contrast to other cluster.

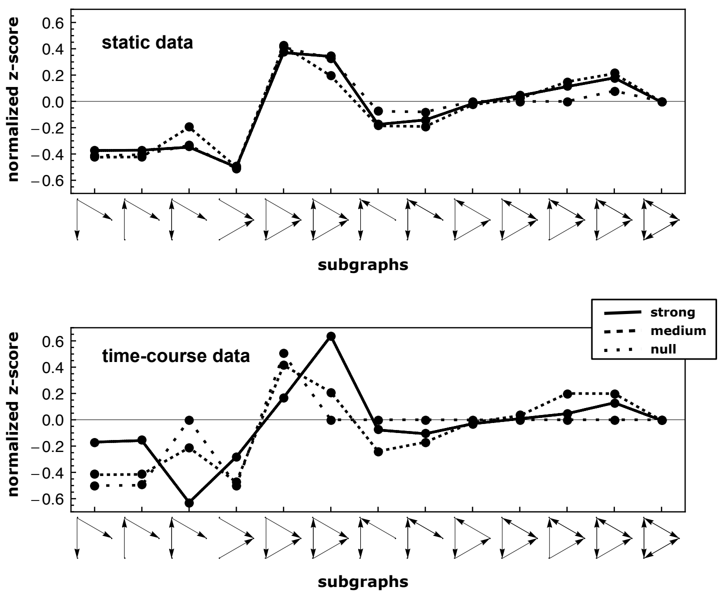

Figure 4. Normalized z-score profiles of all 13 possible directed triads. For the two expression data sets (top: static data, bottom: time-course data) we calcluate the normalized z-score profiles for all identified clusters of different subnet usage. The networks of all cluster show the triad significance profile of bacterial regulatory networks (compare with [12]).

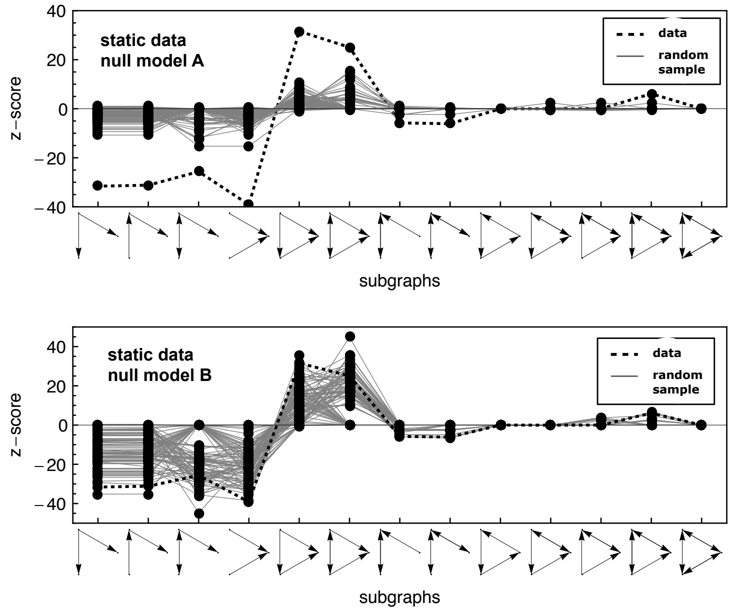

Figure 5. Comparison with null model z-score profiles. For both null models (see main text for details) we find that the feed-forward loop z-score of the 'null' cluster exceeds both null model averages.

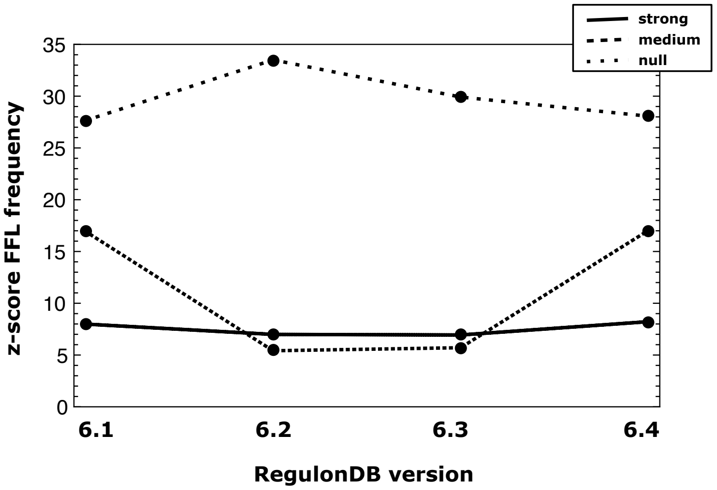

Figure 6. FFL enrichment in the time-course data, using the last four version of RegulonDB (6.1 - 6.4). We find that irrespective of the RegulonDB version used in our analysis, a prominent FFL enrichment in the 'null' cluster appears.

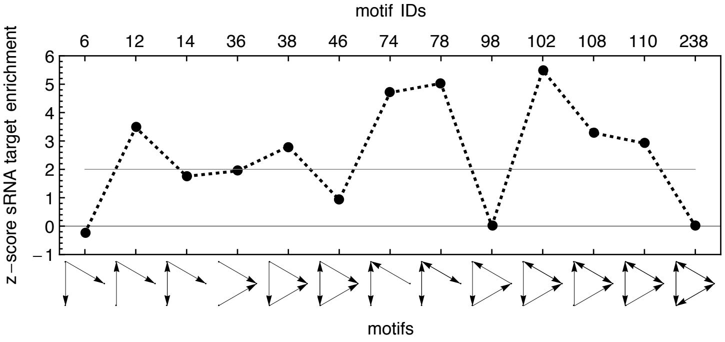

Figure 7. sRNA mediated control on network motifs. Comparing the number of 3-node motifs with at least one sRNA target with randomly sampled sets of targets of the same size, we identify seven motifs with significantly ($z - score \geq 2$) enriched occurrence of sRNA targets.
